# The quality enhancement of action research on primary school English instruction in Chinese rural areas: An analysis based on multimodality

**DOI:** 10.3389/fpsyg.2022.1013021

**Published:** 2022-09-29

**Authors:** Haiyan Zhang, Cunxin Han, Hongyan Ma, Liusheng Wang

**Affiliations:** ^1^School of Foreign Languages, Jimei University, Xiamen, Fujian, China; ^2^School of Foreign Studies, Nantong University, Nantong, Jiangsu, China; ^3^Teachers College, Jimei University, Xiamen, China

**Keywords:** action research, quality enhancement, multimodal discourse analysis model, primary school English teaching, rural areas

## Abstract

This study investigates the influences of action research on primary school English instruction from five dimensions in the classroom, *viz.*, types of questions, language errors, gestures, facial expressions, and interpersonal distance. Four English teachers’ 9 real classroom teaching videos before and after action research are collected and annotated by using ELAN software. The results show that primary school English teachers in Chinese rural areas prefer closed questions to open questions; They make some language errors; Deictic gestures are the most common gestures used, while metaphoric gestures, beat gestures and iconic gestures are rare; Teachers have the same preference for three types of facial expressions, and teachers’ serious expressions accounts for most of the time; They seldom keep an intimate distance or personal distance from their students. Second, Action research is effective to motivate teachers in rural areas, who make great progress in all five dimensions after AR: more open questions are asked; pragmatic errors and grammatical errors are reduced; deictic gestures increase; apathetic decrease, and more intimate distance is exhibited in the interpersonal distance dimension. Third, teacher’s English teaching competence is partly transferrable to her future professional development, and this is also the long-lasting effect of AR.

## Introduction

English, as a foreign language, is a compulsory course in most Chinese primary and middle schools since the issue of the National English Curriculum in 2001, in which, English is required to be learned when students are at the 3rd grade in primary schools and in some developed areas, English can be arranged as a compulsory course at the 1st grade. Because of the requirement for a large amount of the primary school English teachers in the whole country, there is a great difference between the English teacher’s competence in developed areas and the rural areas. Teachers with higher English teaching competence would like to work in the developed areas for the high salary, good educational environment, etc., and the teachers with low English teaching competence have to work in rural areas for the no serious competition and not high requirements of the parents and students. For example, Primary school English teachers in fast-developed urban areas, such as Shanghai and Beijing, are required to have at least a bachelor degree, even a master or doctor degree. Whereas, the majority of teachers in rural areas only get tertiary school education; only a few of them have attended the university. That means they do not have qualified educational backgrounds and teaching capabilities to teach students. Secondly, teachers have few opportunities for further training to level up their teaching ability. So most of the teachers still use the traditional method to teach English. For example, in vocabulary teaching, words are only uttered repeatedly in a chorus and the Chinese corresponding meanings are given to students directly. Students take notes, recite and spell the words, and then do the dictation practice. These traditional teaching methods are outdated and cannot meet the demands of new National Curriculum Reform, which emphasizes on the improvement of the students’ linguistic competence, cultural awareness, thinking disposition and autonomous learning (Ministry of Education, 2022). As a fact, English teaching in rural areas is far from satisfactory. Most of English teachers there are not education majors or English education majors. This means they do not have enough qualifications or English training during their pre-service stage. Furthermore, due to lack of opportunities for professional development, it is hard for these rural school English teachers to update their teaching concepts and improve their teaching quality.

In Chinese English teaching, most teachers prefer the traditional grammar-translation method, in which, English teaching is a kind of knowledge imparting, and the process of students’ English learning focuses more on memorization of new words and grammatical rules rather than applying these new words and grammatical rules creatively or voluntarily experiencing another culture, which makes their English learning more like knowledge accumulation. English teachers in rural areas greatly need opportunities to facilitate their professional development. According to the Organization for Economic Co-Operation and Development (OECD), the development of teachers beyond their initial training can update individuals’ skills, attitudes and approaches in light of the development of new teaching techniques and objectives, new circumstances and new educational research and help weaker teachers become more effective (OECD, 1999). Teachers can participate in many types of activities for professional development, such as courses/workshops, education conferences or seminars, qualification programme, observation visits to other schools, participation in a network of teachers, individual or collaborative research, and mentoring and/or peer observation and coaching (OECD, 2009). Among them, mentoring and peer observation and coaching are applied in this study to help primary school English teachers identify their problems in English teaching. Then, these teachers are instructed to design an appropriate lesson plan and apply this plan to new instruction. The final step is to help these teachers reflect on their teaching to facilitate their future development, which relies on the process of action research (AR).

Kemmis et al. (1982) argues that AR is an ideal method to help people reflect on themselves. It can be employed to motivate human’s awareness and practice in various fields, such as sociology, education, etc. In traditional classroom teaching, teachers focus on students’ performance and behaviors, seldom reflect and monitor whether their own teaching performance and process were appropriate (Huang and Ling, 2016). The teachers’ role in traditional teaching was to control the students, instead of controlling themselves, which inhibit the improvement of teaching. By adopting AR, teachers could consciously conduct their own reflection and synchronize with both teaching practice and experiments. With the assistance of “action” and “research,” new teaching ideas and methods will develop (Nunan, 1990). Only when these teachers experience the entire recycling process can they realize how to improve their personal teaching quality. This study endeavors to investigate features of rural primary school English teachers’ classroom teaching before and after AR, whose effectiveness and impact are also examined.

## Literature review

### Action research

According to Kember (2000), AR has the following five features: teacher-student interaction, improved practice, continuous circulation and spiraling, self-reflection, and direct teacher participation. Cohen et al. (2000) claim that “authenticity, small range, intervention, observation and thinking” are the features of AR. McNiff (1988) extracts four features (participant, cooperative, systematic, and experimental) of AR and demonstrate five procedures, that is, finding teaching problems, making feasible plans, taking plans into action, gathering data, and finding new problems. Likewise, Altrichter and Al (1990) proposes seven steps of AR based on McNiff’s procedures, and make the process more specific. Although researchers opt for different procedures under various research circumstances, they believe four procedures are essential in AR, i.e., planning, implementing, observing, and reflecting.

Kember (2000) classified quality schemes into quality assurance and enhancement. The former is a top-down quality control process, and the latter is a bottom-up quality enhancement process. The very essence of quality enhancement is improvement, which encourages better teachers toward higher quality and more innovative practices. Based on the concept of quality enhancement, it is necessary for teachers in Chinese rural areas to apply AR in their instruction, as Chinese Confucius etiquette expert Dai said 2000 years ago: “When he learns, one knows his own deficiencies; when he teaches, he knows the difficulties of learning. After he knows his deficiencies, one is able to turn round and examine himself; after he knows the difficulties, he is able to stimulate himself to effort” (Dai and Legge, 2016). Realizing one’s deficiencies is the first step towards future development, and only when he takes some measures or performs some actions can his teaching quality be enhanced.

There is a new trend in the study of AR, *viz.* researchers change their focus from the traditional study of AR, which emphasizes the self-role in the whole process, to the application of various kinds of AR to improve teachers’ professionalism, such as collaborative AR approaches (Dulfer et al., 2021), professional learning networks (PLNs; Poortman et al., 2022), professional learning communities (Long et al., 2021) and quality teaching rounds (Bowe and Gore, 2017). All these new models of AR emphasize the importance of collaboration among teachers or teachers with other professional communities. Sometimes teachers’ shortcomings or deficiencies cannot be realized by themselves; in this case, others’ participation and collaboration will play a very important role in improving their professionalism. With the rapid development of modern educational technology, multimodality theory has been employed more to analyze the teachers’ instructions to help them have a direct understanding of their own teaching. A number of scholars explored this area. Romney. (1997) conducted two semesters of AR on students in poverty-stricken areas, trying to introduce the concept of “community of practice” that appeared in other teaching fields. It proves that learning cooperatively is advantageous to enhance their French translation and communication skills in translation teaching. Burns (1999) conducts AR on three teachers in relative undeveloped areas. He finds that students have become more active and confident in language learning after AR and it is applicable to take AR to narrow the education gap. Crookes and Chandler (2001) use AR to study students’ own teaching in poverty-stricken areas. By comparing the conversations between students before and after AR in 2 weeks, it finds that their conversation skills are improved, and their spoken language is more authentic and native.

AR in English teaching has developed rapidly in China and begun to prevail in the undeveloped areas. Guo et al. (2002) carried out two academic years of teaching practice in rural areas and finds students’ oral performance has made great progress. Wang and Wang (2013) find after a series of cyclic measures of AR, it not only enhances students’ listening ability, but also arouses teachers’ sense of responsibility for scientific research in poverty-stricken areas. Yang and Li (2017) conducted an AR in students’ English writing in rural areas and reveal that most students (62.5%) think their writing level has been improved after AR. Zheng and Chen (2000) discusses the practice of AR in reading teaching in undeveloped areas and find students’ reading capability enhanced and teachers’ interest of scientific research aroused. These studies show that AR in poverty-stricken areas have lots of advantages for English teaching, including reading, listening, writing and speaking. As can be seen that AR has become an effective means to facilitate teachers’ professional development. It can be used not only to solve problems in specific teaching, but also assist teachers to grow into reflective teachers.

### Multimodal discourse analysis

Multimodality can be understood as a theory, a perspective, a field of enquiry or a methodological application (Jewitt, 2006) that applies new theoretical and methodological frameworks to analyze communication that integrate modes or nonverbal signs beyond verbal language (Kress and van Leeuwen, 1996; O’Halloran, 2004), and these signs are mutually interactive with each other, expressing meaning together with language signs. Multimodal discourse combines a wide range of semiotic modes in communicative activities (Van Leeuwen, 1999). Gu (2007) claims that it is rare for human beings to interact with others with a single method or in a single way. Cope and Kalantzis (2009) point out that communication is intrinsically multimodal and that linguistic, visual, audio, gestural and spatial modes of meaning are becoming increasingly integrated into everyday media and cultural practices. Mode is central to multimodality, as it is the selection from linguistic, visual, gestural, audio and spatial semiotic resources and how these are combined that provides the means for communication for making meaning (Kress, 2010).

With the development of multimodality theory, an independent academic field of multimodal discourse analysis (MDA) is formed, beginning with Barthes’ decomposition of images and language to express meaning (Barthes, 1994), which is regarded as an important theoretical basis for MDA. Kress (2003) researches on the construction of MDA and tries to design multimodal analysis software. O’Halloran (2004) discusses the multimodal symbols in teaching discourse. Royce and Terry (2015) explain the complementarity and the multimodal coordination of various modalities in second language teaching.

Some empirical evidence demonstrates a significant role of MDA in teacher’s performance or practice. Recently, Qin and Wang (2021) implement a case study including two-winner teachers utilizing their multimodal ensembles of communicative modes to engage students during classroom lead-ins, involving facial expression, gaze, distance, spoken language, print, gesture, head movement, and posture. EFL teachers’ high multimodal competence plays a decisive role in performing classroom lead-ins, and different lead-ins strategies affect the different orchestration of communicative modes (Qin and Wang, 2021). Taking the teacher’s facial expression as an example, the role of teachers’ facial expressions in students’ learning is helpful to improve online teaching (Wang, 2021). Teachers’ facial expression is a direct indicator showing their attitudes toward students (Qin and Wang, 2021). So, teacher’s facial expression could affect students’ learning attitude, motivation, and the qualities of interaction between teacher and students. Chinese EFL students’ affective learning is largely influenced by teacher-student rapport and teacher support (Sun and Shi, 2022). Higher-level actions as one of mediated actions proposed by Norris, could support MDA. Higher-level actions are the sum of chains of different single actions (Norris, 2004, 2020), and higher-level actions make the actions interdependent (Zhang and Wang, 2016).

**Figure 1:**
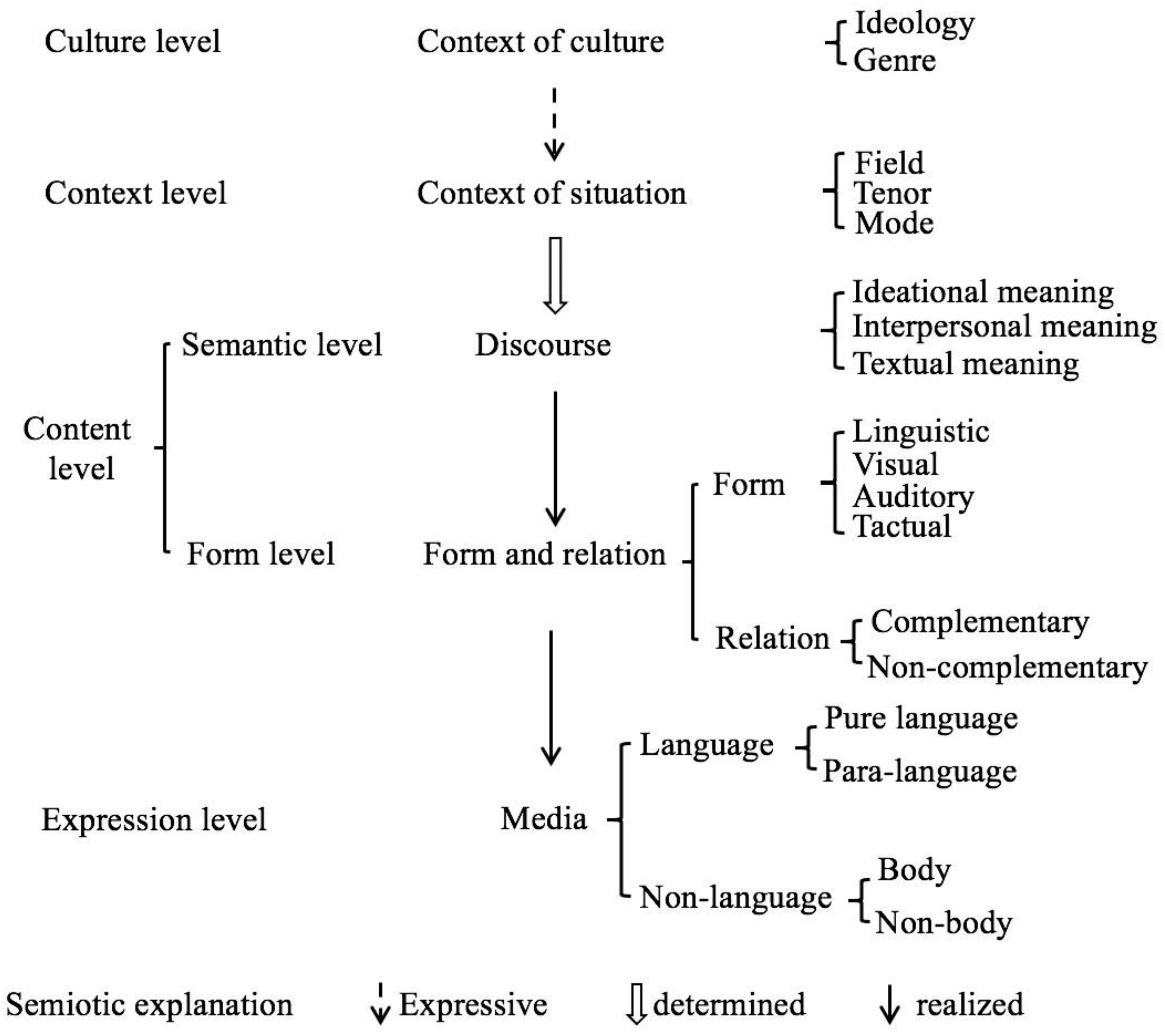
Framework of MDA (Zhang, 2009).

Zhang (2009) proposes an outline of the multimodal discourse analysis model (MDAM; see Figure 1), which includes four levels—culture level, context level, content level, and expression level. These four levels can be represented by the contexts of culture, context of situation, discourse, form and relation, and media. This framework suggests an import role of multimodal discourse analysis in the English teaching class.

It is not until the recent years that Chinese scholars apply MDA to teaching English in rural areas. For instance, Hu (2017) uses multimodal teaching methods on junior high school students in poor areas. Yang (2019) also conducts an empirical study to put MDA into vocabulary teaching to rural primary school students. This method alters their negative attitudes and make their vocabulary learning more efficiently and fruitfully. Xia (2020) demonstrates that MDA - assisted teaching is advantageous for the timely and delayed vocabulary learning compared with traditional teaching approach. Wang (2020) shows that multimodal teaching is effective for students in undeveloped areas to build a large vocabulary. And it is suggested that teachers should integrate different modes to assist teaching in order to enhance both teaching and learning. However, seldom previous studies are about the longitudinal study on the effects of Action Research on English teachers in rural areas from the perspective of multimodality. Most of the researches concern on the status quo of the English classroom teaching, which belongs to the horizontal study. This study is a breakthrough in this aspect.

## Purposes of the present study

This study tries to explore the current situation and problems in the teaching of English in the poverty-stricken areas of China, as well as examine the influence of action research on English teaching. Based on Zhang’s MDA, three questions are proposed in this study: (1) What are the multimodal features of four primary school English teachers’ classroom teaching in rural areas? (2) Can AR effectively enhance the quality of English teaching in rural areas? If so, in which aspects? (3) Can teachers’ English teaching competence be transferrable after AR? Which aspects of multimodality can be transferred? Which aspects still need to be focused on? The answers to these questions would shed light on primary school English teaching in Chinese rural areas. In addition, it is hoped that the study can help teacher education institutions and teacher training centers apply appropriate AR to improve English teachers’ teaching competence.

## Methodology

### Research methods

Two research methods, observation and action research, are applied in this study to reveal the overall features of teaching. By observing English teaching videos, qualitative results are collected. And AR is applied to acquire evident and convincing quantitative data. Thus, this study integrates two methods to draw more reliable research results.

#### Observation

Four teachers’ teaching videotapes in class are collected and observed repeatedly, which provides a large amount of information concerning human’s various senses. No direct interference to students during video observation is the major merit of this method, so that the real classroom teaching is able to be observed. Sometimes, one clip of teaching video is even played more than 10 times in order to observe carefully. Repeated observation of the teaching videos has made sufficient preparations for future analysis and supplement for data. Therefore, it is undoubted that the observation method provided powerful support in this study.

#### Action research

**Figure 2:**
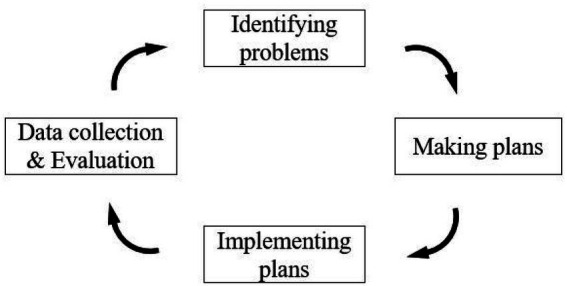
Action research cycle.

McNiff’s AR cycle is followed in this research (1988), which is composed of four main procedures: identifying problems in teaching, making plans to tackle problems, implementing plans, as well as collecting and evaluating data (see Figure 2).

### Participants

This study takes 4 female primary school English teachers’ teaching as the main subject. These teachers work in the southwestern part of China, which belongs to a poverty-stricken area before 2020, this is an important year which marks the alleviation of the poverty in whole China. Four primary school English teachers’ nine 45-min classroom teaching videos are studied in this research. These four teachers are coded as Teacher A, B, C, and D, aged M = 31.75, mean of years of teaching M = 42.00. The teachers’ personal profile and the teaching topics are shown in Table 1. These nine videos include four teachers’ teaching videos before AR, four teachers’ teaching videos immediately after AR, and Teacher A’s third teaching recording after 2 months. Four teachers’ teaching videos before AR are used to find the features of their English teaching, and four teachers’ teaching videos after AR are contrasted with the first recording of their teaching to investigate the influences of AR. When confronted with new teaching topics 2 months after AR, Teacher A designs a teaching plan and carry out teaching practice by herself for the third teaching session. It aims to test whether Teacher A’s teaching competence acquired in AR is transferrable to future teaching and explore the deficiency that still remains in English teaching after AR.

**Table 1:** The teachers’ personal profile and the teaching topics.

Teacher’s Surname	Coding No.	Years of Teaching	Age	Before AR	After AR	2 Months after AR
Chen	A	10	31	A1 What Would You Like	A2 What Would You Like	A3 Work Quietly
Liu	B	12	37	B1 Look at Me	B2 Look at Me	
Wu	C	12	30	C1 Let us Eat	C2 Let us Eat	
Shu	D	8	29	D1 This is a Big Bed	D2 This is a Big Bed	

### Research procedures

After collecting the first teaching videos, four teachers participated in AR to improve their teaching. (1) Four English teachers and their advisors (one professor majoring in English Curriculum and Pedagogy and one excellent Primary School English teacher trainer) observe teaching videos and tried to determine the problems of their English teaching, including incorrect pronunciation, inappropriate teaching design, classroom management, and assignments. (2) With the guidance of the two advisors, the four English teachers realize these problems, and they endeavor to develop plans to improve their teaching. (3) New plans are put into practice, and they teach the same content in the second class. (4) The two advisors and the four teachers evaluate the second teaching videos and check whether these plans are valuable and whether their teaching is improved. (5) Teacher A voluntarily design a third teaching plan for a new lesson after 2 months of AR, and the third video is evaluated by the advisors to determine whether the teacher’s teaching ability improved through AR is transferable to new teaching sessions. In total, nine videos are collected for analysis to determine the teaching features of these four English teachers’ teaching and the effect of AR on their teaching from the perspective of multimodality.

This research focus only on the visual and auditory modes. According to Zhang’s MDA, visual and auditory modes in teaching are separated (see Table 2). The auditory mode mainly includes the types of questions and language errors, and the visual mode mainly includes the teacher’s gestures, facial expressions, and interpersonal distance.

**Table 2:** Modes annotated in teaching.

Auditory Modes	Type of Question	Closed Questions (CQ)
		Open Questions (OQ)
	Language Errors	Grammatical Errors (GRE)
		Pragmatic Errors (PRE)
		Phonetic Errors (PHE) Lexical Errors (LE)
Visual Modes	Gestures	Deictic Gesture (DG)
		Beat Gesture (BG)
		Iconic Gesture (IG)
		Metaphoric Gesture (MG)
	Facial Expression	Smile (SM)
		Seriousness (SE)
		Apathetic (AP)
	Interpersonal Distance	Intimate Distance (ID)
		Personal Distance (PD)
		Social Distance (SD)

This study uses the multimodal analysis software ELAN and data statistics software SPSS to analyze the collected videos. Each video is annotated to indicate the above five major factors, and there are 16 layers in total.

## Results

### Type of questions

Before and after AR, all the teachers ask many questions in their English classes. In this study, open questions (OQ) and closed questions (CQ) are analyzed. Whether before or after AR, the percentage of CQ was more than 60% (see Table 3). It showed that teachers tended to ask more CQ than OQ even after AR. According to Table 3, the proportion of OQ for the four teachers after AR increased significantly, and all of them had reached Borich’s standard. If the teaching content emphasized a low level of complexity, the best ratio of CQ to OQ was 70:30, and if the class content emphasized a high level of complexity, the best ratio between the two was 60:40 (Borich., 2002). Table 3 shows that after AR, the ratio of CQ to OQ of Teacher A was 69:31, the ratio of Teacher B was 68:32, the ratio of Teacher C was 62:38, and the ratio of Teacher D was 63:37. Among them, Teacher C and Teacher D changed most significantly. The proportion of OQ for Teacher C in the second English teaching increased greatly from 26 to 38%. That means that in the total 47 questions, there were up to 18 OQs.

**Table 3:** Description of types of questions before and after AR.

		A		B		C		D		Standards	Total
Questions	Stages	f	%	f	%	f	%	f	%	%	f	χ^2^
CQ	Before AR	50	76	42	78	37	74	34	85	60–70	163	3.48
	After AR	54	69	42	68	29	62	44	63	60–70	131	
OQ	Before AR	16	24	12	22	13	26	6	15	30–40	47	12.45^***^
	After AR	24	31	20	32	18	38	26	37	30–40	88	

The same applies below. f: Frequency; %: Percentage of Frequency.

* *p *<0.05;

** *p *< 0.01;

*** *p *<0.001.

The Chi-square test showed that (Table 3) the frequency of OQ before AR was extremely significantly smaller than that after AR, χ^2^ (1) =12.45, *p* < 0.001, while there was no significant difference in CQ frequency before and after AR, χ^2^ (1) =3.48, *p* > 0.05. The results indicated that AR is effective in altering the number of OQs in class.

**Example 1** (Transcription of Teacher C’s question proposal before AR)

T: There is a mouse behind the computer. Is this right or wrong?

S: Yes.

T: Does John live near the natural park?

S: Yes.

T: Are there some pencils and crayons on the floor?

S: Yes.

**Example 2** (Transcription of Teacher C’ question proposal after AR)

T: Where is the mouse?

S: It’s behind the computer.

T: Where does John live?

S: He lives near the natural park.

T: What can we find on the floor?

S: There are some pencils and crayons on the floor.

As can be seen from these two examples, before AR, Teacher C proposes a series of CQs in the while-reading part to check whether students have a better understanding of the text. All these CQs are less complex and less demanding for students, as they require only answers of “Yes” or “No” and do not require much speaking. In contrast, after AR, OQs are more difficult than CQs, which facilitates students to think, organize and output language to answer the teacher’s open questions. The students are able to express themselves in long sentences instead of just answering “yes” or “no.” Student behavior and English usage is determined by the teacher’s question type, and proper question types can provide students with more opportunities to learn English in practice.

### Language errors

Language errors can be divided into phonetic errors (PHE), grammatical errors (GRE), lexical errors (LE), and pragmatic errors (PRE) in second language learning (Corder, 1974). Through annotation and analysis, no LE appear in any of the teaching sessions, so only PHE, GRE, and PRE are analyzed. As shown in Table 4, comparing the language errors in teaching videos before and after AR, among these three kinds of errors mentioned, the PRE was always the lowest. Most of the teachers made no more than 10 PREs before AR, except Teacher D. After AR, this kind of mistake was reduced to less than 6 for all teachers. This showed that the teachers performed well and did not have much difficulty giving instructions in class. They could make clear and correct instructions. All four teachers had a certain reduction in GE and PRE. However, the reduction in PHE was not obvious (see Table 4). There was still a large amount of PHE after AR.

**Table 4:** Description of errors and gestures before and after AR.

			A		B		C		D		Total
Variables	Dimension	Stages	f	%	f	%	f	%	f	%	f	χ^2^
Errors	PHE	Before AR	36	60	24	57	42	64	47	52	111	2.53
		After AR	42	76	18	56	32	60	44	68	136	
	GE	Before AR	18	30	14	33	16	24	31	34	79	3.92^*^
		After AR	10	18	12	38	16	30	18	28	56	
	PRE	Before AR	6	10	4	10	8	12	14	15	32	8.02^**^
		After AR	3	6	2	6	5	10	3	4	13	
Gestures	DG	Before AR	37	47	42	45	54	68	75	73	170	6.17^*^
		After AR	46	50	48	42	62	68	63	64	219	
	MG	Before AR	28	35	23	24	12	15	21	20	84	0.15
		After AR	32	35	27	23	14	15	24	24	79	
	BG	Before AR	3	4	18	19	4	5	5	5	30	3
		After AR	2	2	26	23	8	9	9	9	45	
	IG	Before AR	11	14	11	12	9	11	2	2	33	0.13
		After AR	12	13	14	12	7	8	3	3	36	

f: Frequency; %: Percentage of Frequency. *p < 0.05; **p < 0.01; ***p < 0.001.

The Chi-square test showed that the frequency of errors in GE and PRE before AR was significantly larger than those after AR (Table 4), GE, χ^2^ (1) =3.92, *p* < 0.05; PRE, χ^2^ (1) =8.02, *p* < 0.01. There was no significant difference before and after AR in PHE, χ^2^ (1) =2.53, *p* > 0.05. This showed that AR affected the rate of GE and PRE. However, it did not substantially affect the teachers’ in-class PHE.

To acquire more exhaustive insight into the issue concerned, all four teachers were asked to reflect on their teaching after class. Through their reflection excerpts, the factors that hindered their improvements can also be analyzed.

[**Teacher A**] “Even though I have taught English for many years, I still make some mistakes in class. For example, I can’t help myself using Chinese to explain some words and sentences. I know it cannot provide students with a foreign language learning environment, but it is hard for me to explain some words in English. My bad spoken English and students’ comprehension make me use Chinese frequently. My English is prone to be worsened by the Chinese grammatical rules. Pronunciation errors often appear in my class. Even if I have a solid belief in improving my pronunciation, I cannot correct these pronunciation errors in a short time because I have pronounced some words wrong for a long time and no one help me correct them.”

[**Teacher B**] “I know there are still many problems existing in my teaching. I used to teach Chinese in this school and changed to teach English four years ago when English teachers are badly needed in W county. I’m not confident about my English pronunciation. English is not my major, and I haven’t learned pronunciation systematically. Some professional items, such as some intonation, stress, and liaison, are hard for me to imitate. I hope I can have an opportunity to polish my English speaking in the future.”

[**Teacher C**] “My English pronunciation is terrible. I assert that it is mainly affected by our dialect. I often speak English with the accent of our dialect unconsciously. Even though some teachers remind me that I have pronounced incorrectly, I cannot correct them by myself. It seems unavoidable for me to speak English with the influence of dialect.”

[**Teacher D**] “I often make some mistakes in English speaking. And, I’m confused about how to improve it and change this situation. There isn’t sufficient training for me to learn from some excellent teachers. I can hardly have opportunities to watch other teachers’ classes in W county. Although there exist assistant devices, such as multimedia and the internet, I seldom have the energy and time to devote myself to improving my teaching. Because I have to take care of my family and even do some farming work to make a living besides school teaching.”

From the excerpts of the four teachers’ self-reflections above, the reasons why there are still so many pronunciation errors after AR can be found in the following three aspects. First, most English teachers were not English majors, and they taught other subjects, such as math or Chinese before they were transferred to teach English. They know little about liaison, stress, or intonation, so it is difficult for them to pronounce correctly. Second, Chinese grammar, pronunciation, and thinking all influence English teaching and learning, especially their dialect, which has a negative impact on pronunciation. Last, except for GE and PRE, which can be corrected easily, PHEs are entrenched and can be difficult to correct. For example, the teachers still became confused about the pronunciation of the vowel [æ] and the consonants [θ], [ð] and [s], [z].

### Gestures

According to Zhang’s MDA, a teacher’s gestures can be divided into the deictic gesture (DG), beat gesture (BG), iconic gesture (IG) and metaphoric gesture (MG). In this study, teachers’ gestures do not change substantially before and after AR. This means that the four teachers still prefer to use a large number of DGs in their teaching, followed by MGs and IGs. After AR, BGs were the least used by the teachers. For example, Teacher C used 54 DGs, 12 MGs, 4 BGs, and 9 IGs before AR. In addition, after AR, in the second teaching session, she used 62 DGs, 14 MGs, 8 BGs, and 7 IGs, indicating little change (see Table 4).

The Chi-square test showed that (Table 4) the frequency of DGs before AR was significantly smaller than that after AR, χ^2^ (1) =6.17, *p* < 0.05. There was no significant difference in MGs, BGs or IGs before and after AR; for MGs, χ^2^ (1) =0.15, *p* > 0.05; for BG, χ^2^ (1) =3.00, *p* > 0.05; for IG, χ^2^ (1) =0.13, *p* > 0.05. It is reasonable to conclude that AR influences DGs instead of the other three kinds of gestures.

As an example (see Table 4), Teacher C did not greatly change her gestures. She still used a large number of DGs while teaching. However, there were fewer of other kinds of gestures. DGs are useful in assisting teachers to acquire students’ attention quickly in class. For example, she often uses DGs to point to the blackboard, the PPT, and students who are asked to answer questions. It minimizes the class time required for tasks other than language instructions and periodically motivates students to focus on the class. However, the other three gestures should also be encouraged to benefit teaching. For instance, when encountering unfamiliar vocabulary for students, gestures can be used to explain the meaning instead of simply telling students the Chinese meaning. In the second teaching session, when teacher C explained the phrase “far away from,” she only translated it into Chinese, and the students wrote down the meaning in their notebooks. By explaining this phrase vividly, she could stretch her arms and open her hand to show its meaning while leading the students to read the phrase with a rhythm. With the assistance of MGs and BGs, she could explain the phrase easily. Students will remember this phrase quickly in class. Many aspects of applying gestures need to be perfected. The reasons gestures cannot be applied flexibly by teachers can be seen from the reflection excerpts of teacher C.

The reflection excerpts of teacher C are as follows: “I was not aware of the meaning of body language before. And, through AR, I began to think about how to use body language, such as gestures and postures, to facilitate my teaching. I am familiar with DG, and I often use DG to attract people’s attention. However, gestures require a certain accumulation in a long term. I have difficulties in accumulating and applying other gestures to help my teaching appropriately.”

Gestures cannot be applied without continuous accumulation. In addition to DG, a half-year AR is not sufficient to make difference from the other three gesture applications in English teaching.

### Facial expression

Compared with the three aspects discussed above, the outcomes of interpersonal distance are lower than expected. The apathetic (AP) still accounts for the largest percentage, up to 60% of the entire class, while the percentage of smiling and serious expressions remained steady (see Table 5). For example, even in the second teaching, 67% of the teaching time in teacher D’s class is apathetic (AP), with smiling (SM) and serious expressions (SE) accounting for only 15 and 17%, respectively.

**Table 5:** Description of facial expression and interpersonal distance before and after AR.

			A		B		C		D			
Variables	Dimension	Stages	Duration	%	Duration	%	Duration	%	Duration	%	M	Z
Facial	SM	Before	393	16	311	15	765	34	697	27	541.5	0
		After	645	25	411	17	715	36	389	15	540	
	SE	Before	393	16	415	20	135	6	310	12	313.25	0.73
		After	619	24	460	19	159	8	441	17	419.75	
	AP	Before	1,671	68	1,349	65	1,350	60	1,574	61	1,486	1.83∆
		After	1,315	51	1,549	64	1,112	56	1739	67	1428.75	
Distance	ID	Before	516	21	187	9	248	11	387	15	335	-1.826∆
		After	645	25	532	22	477	24	909	35	641.5	
	SD	Before	1,180	48	1,224	59	1,530	68	1,419	55	1338.25	−1.095
		After	516	20	1,016	42	1,112	56	857	33	875.25	
	PD	Before	762	31	664	32	473	21	774	30	668.25	−1.826∆
		After	1,418	55	871	36	397	20	831	32	878.75	

∆*p* < 0.1, the unit of time is second.

The Wilcoxon test showed a marginally significant difference in AP before and after AR (Table 5), Z = −1.83, *p* = 0.068, while there was no difference in SM or SE before and after AR, *p* > 0.05. The results illustrated that the teachers did not change significantly in SM or SE but did change in AE after AR. The teachers shortened the AP time during teaching, while SE and SM varied little.

There are two reasons for this phenomenon. First, the usage of facial expressions in class depends on various factors, such as the teaching style, content, and students’ reactions in class. These four teachers did not change their facial expressions flexibly to suit the situation. For example, when explaining difficult topics and skills, they should try to smile to assist students in feeling that the teacher is easy-going and less stressed; when responding to students’ answers, teachers can smile to encourage students regardless of whether the answer is correct or not; when some students become distracted in class, teacher can change their expressions to express seriousness to warn the students to return their attention to class and concentrate on the teacher.

### Interpersonal distance

In terms of interpersonal distance, teachers started to change their positions in the classroom after AR. Before AR, four teachers maintained SD most of the time. After AR, the proportion of ID and PD time increased greatly, indicating that AR is effective in reminding teachers to modify their distance from students in class (see Table 5). More specifically, the ID time of teacher A increased from 21 to 25%; that of teacher B climbed from 9 to 22%; that of teacher C rose from 11 to 24%; and that of teacher D jumped from 15 to 35%. However, the SD time of teacher A decreased from 48 to 20%; that of teacher B decreased to 42% from 59%; that of teacher C decreased from 68 to 56%; and that of teacher D decreased from 55 to 33%. These statistics reflect that those teachers tended to stand close to students and avoided standing on the platform alone.

The Wilcoxon test showed a marginally significant difference in ID and PD before and after AR (Table 5), *p*s < 0.1. There was no significant difference in SD, Z = −1.095, *p* > 0.05. Therefore, AR can exert positive effects on teachers’ PD and ID in teaching. Even after AR, the teachers did not have many changes in their IDs. It is worth examining the cause of the difference.

As an example (see Table 5), teacher B’s ID changed greatly. The time exhibiting SD for teacher B decreased from 59 to 42%. She spent up to 36% of the teaching time exhibiting PD. She also increased the time spent exhibiting ID with students from 9 to 22%. In the teaching after AR, when a student was distracted during the class, teacher B changed from ID to PD. She slowly walked down the platform and approached the student, and the student immediately changed his behavior and returned to concentrating on the class. Furthermore, when dictating words, she walked and read in the aisle, adjusting the speed at which she dictated words. In this situation, the students felt nervous and received a kind of silent warning through ID. She also made full use of changing distances in the class. Teacher B also walked among the students, observed group discussion, and offered some help to students in groups. Before AR, teacher B kept a watchful eye on the students who sat in the front of the class (the first three rows) and often invited these students to answer questions or have teaching interactions. However, in the second teaching session after AR, with the changes in interpersonal distance, teacher B stood close to all the students who sat in the front of the classroom. She considered the entire class and let all students join in the class interaction, which stimulates learning motivation.

### Teacher A’s English teaching 2  months after AR

To test whether the teaching competence they acquired through AR can be transferrable in their future teaching, the third teaching session of teacher A was analyzed and compared with her previous teaching sessions. Two months after AR, Teacher A records her third English teaching session. The teaching content differs from those of the previous two teaching sessions, and she designs and organizes all of the teaching content. Therefore, by analyzing her third teaching video and comparing the data from all three rounds of teaching, the aspects that still need to be improved can be identified. The data in Tables 6, 7 reveal that significant changes occurred in the types of questions asked, language errors made and gestures used; however, ID and facial expressions showed little change.

**Table 6:** Frequency changes in Teacher A’s three teaching sessions.

		A1		A2		A3		χ^2^	*p*
		f	%	f	%	f	%		
Types of Questions	CQ	50	76	54	69	30	56	7.403	0.025^*^
	OQ	16	24	24	31	24	44	2.000	0.368
Language Errors	PHE	36	60	42	76	12	67	16.800	0.000^***^
	GE	18	30	10	18	6	33	6.588	0.037^*^
	PRE	6	10	3	6	0	0	1.000	0.317
Gestures	DG	37	47	46	50	37	37	1.350	0.509
	MG	28	35	32	35	42	42	3.059	0.217
	BG	3	4	2	2	4	4	0.667	0.717
	IG	11	14	12	13	17	17	1.550	0.461

*p < 0.05; **p < 0.01; ***p < 0.001.

**Table 7:** Time changes in Teacher A’s three teaching sessions.

		A1		A2		A3	
		Time	%	Time	%	Time	%
Interpersonal Distance	ID	516	21	648	25	708	23
	SD	1,180	48	516	20	677	22
	PD	762	31	1,416	55	1,540	50
Facial Expression	SME	393	16	645	25	831	27
	SEE	393	16	619	24	924	30
	AE	1,671	68	1,315	51	1,324	43

The unit of time is second.

After AR, Teacher A made significant changes in two dimensions of English teaching. First, she used an increasing number of OQs (see Table 6): χ^2^ (2) =7.403, *p* < 0.05. This means that Teacher A began to consider an appropriate ratio of OQ and CQ and increased the quality of the questions, inspiring the students to reflect. By observing the videos, it is easy to see that the students are more enthusiastic to think and organize answers themselves instead of answering simple “Yes” or “No” questions. Even though students may occasionally answer incorrectly, they are brave in answering questions from different perspectives and showing their own opinions. Second, three types of language errors, namely, GE, PHE, and PRE, were reduced to a certain extent (see Table 6). The number of PREs in the third teaching session decreased to zero; the number of PHEs decreased to 16, and the number of GEs decreased to only 6. For PHEs, (χ^2^ (2) =16.800, *p* < 0.0001), and for GEs, (χ^2^ (2) =6.588, *p* < 0.05). This means that Teacher A exhibited significant differences in PHEs and GEs. PREs were always the least common of these three errors, which did not fluctuation substantially in the three teaching sessions. This proves that AR contributes greatly to reducing PHEs and GEs.

There are some aspects that require improvements after AR. First, in the dimension of gestures, the outcomes are far from expected (see Table 6, 7). For DGs, χ^2^ (2) =1.350, *p* > 0.05; for MGs, χ^2^ (2) =3.059, p > 0.05; for BGs χ^2^ (2) =0.667, *p* > 0.05; and for IGs, χ^2^ (2) =1.550, *p* > 0.05. They all revealed that Teacher A had a significant difference in gestures. Therefore, AR did not appear to have much influence for teacher A in this dimension. Second, ID and facial expression did not change substantially (see Table 7). Compared with the previous two teaching sessions, teacher A did not change significantly in ID. She primarily maintained PD from students and less ID. In the third teaching session, AE accounted for most of the teaching time, and facial expressions did not change frequently. This may be connected with teacher A’s serious teaching style and the personal characteristics of effeminacy.

By comparing Teacher A’s three teaching sessions, there are still some aspects that need to be improved. She had insignificant differences in three dimensions: gestures, ID and facial expressions. This illustrates that even after AR, teachers still need to continuously reflect on their teaching. It is necessary to conduct more AR to improve English teaching.

## Discussion

The central issue of this study was what were the multimodal features of four primary school English teachers’ English instruction in rural areas as well as whether AR can effectively enhance the quality of English instruction there. We probed into these questions from two aspects: the teachers’ choice of multimodal ensembles in their English teaching and the quality enhancement of action research on their English teaching. Our findings corroborate the previous point of view that classroom teaching is a multimodal experience that happens through orchestration of spoken language and an array of other communicative modes, such as gesture, gaze, and facial expression (Kress et al., 2005; Jewitt, 2006; Peng, 2019; Lim, 2021; Qin and Wang, 2021). The ensemble of the actions, or the sum of chains of different single actions, belong to the higher-level actions (Norris, 2004, 2020), which make the actions interdependent (Zhang and Wang, 2016). Our findings also reveal that AR is a useful method to improve teachers’ professional development, just as Wallace (1998) mentioned an “empowering procedure,” or quality enhancement (Kember, 2000).

From the pre-action research study, it can be found from the teachers’ classroom multimodality analysis that (1) teachers preferred closed questions (CQs) to open questions (OQs) in English teaching. They asked a large number of CQs and could not come up with creative and high-quality OQs to motivate students to think deeply. The closed questions they asked have definite answers, which were used to test the students’ remembering and reciting of English knowledge; (2) all teachers made some language errors while teaching, including grammatical errors (GEs), pronunciation errors (PREs), and phonetic errors (PHEs). Among them, PHEs appeared the most often. They often made mistakes when pronouncing the vowel [æ] and the consonants [θ], [ð]. All those were the results of the limitation of their English proficiency, as well as the negative transfer of their dialects; (3) Deictic gestures (DGs) were the most common gestures used in teaching, while metaphoric gesture (MGs), beat gesture (BGs), and iconic gesture (IGs) were rare. Teachers were not good at using and changing various gestures to facilitate teaching, which may be the results that those teachers were lack of describing things in English, consequently, the corresponding gestures were seldom used. They used deictic gestures frequently to pointing to the words, or asking students to answer questions; (4) teachers had the same preference for the three types of facial expressions. They preferred serious expressions (SEs) or apathetic (APs) to smiling (SMs) and could not change their facial expressions according to the teaching contents and situations, which may also the results of their low competency in English teaching. When people feel comfortable with a message, they would be able to relax more (van Dijk et al., 2020); (5) when comparing the proportion of the three types of interpersonal distance, teachers exhibited Social Distance (SD) most of the time. They seldom exhibited ID or Personal Distance (PD). This means that they stayed too far away from their students, lacking intimacy and interaction.

Compared with the teachers’ classroom multimodality analysis before and after action research, we can find that for those teachers in rural areas, AR is effective in improving their English teaching. These results can be used to support Burns’ study that AR is applicable to teacher’s teaching in undeveloped areas and narrow the education gap (1999). In teaching practice, most of the teaching behavior is not the result of conscious thinking, nor the attentive selection (Davis et al., 2000). In this study, the function of collaborative AR approach is to increase the teacher-in-need’s attention, to help them focus on some teaching aspects which were often neglected in their normal classroom teaching. Deep collaboration can continuously optimize the problems and strategies, promote the efficiency of classroom teaching, and improve the teacher’s professional development (Qiao, 2018). In this study, after AR, four teachers realized their teaching shortcomings and could made much progress in all five dimensions: more open questions were asked; pragmatic errors and grammatical errors were reduced; deictic gestures increased; apathetic emotions decreased and more intimate distance was exhibited in the interpersonal distance dimension. All these can show that once realized, these shortcomings were easy to be corrected with the facilitative of the collaborators.

The efficient determinant factor in improving teacher education and teacher professional development is to build solid foundation of the teacher’s professional base, including computer literacy (Salman et al., 2022), profound professional skills, cultural skills, educational theories and teaching skills, to enhance the quality of classroom teaching, thus ensuring the “long-term” effectiveness of teaching (Zhu and Li, 2015). When two advisors collaborated with the four English teachers, they not only help them realize the problems of their English knowledge like phonetic errors, grammatical errors and pragmatic errors, but also help them realize the problems in their English teaching approach, their body language, and interpersonal distance, etc. Teacher A’s three teaching videos could vividly reveal that some significant progress has been made in types of questions and language errors; however, she still need to make certain change in the other three dimensions like gestures, interpersonal distance, and facial expressions during the three teaching sessions. As for types of questions, she used an increasing number of OQs than before, which consequently aroused the students’ enthusiasm and learning interests. As for language errors, AR contributes greatly to reduce phonetic errors and grammatical errors. In primary school English teaching, there were not too many words or grammatical structures for students to learn. When advisors pointed out the problems, Teacher A could realize and easily corrected them. The gestures, interpersonal distance and facial expressions all belong to teachers’ teaching style, which was not easy to be corrected. This result could show that teachers’ English teaching competence is partly transferrable to her future professional development, and this is also the long-lasting effect of AR, considering stage 3 was conducted two months after AR. Language Knowledge is easier than teaching style to be transferred.

Finally, limitation and implication would be mentioned. This analysis only focuses on a very small corpus of four EFL primary school English teachers’ classroom teaching, we can only reach some findings which need to be tested by large corpus. However, some pedagogical implications might be drawn from the present research. The curriculum content is expressed, arranged, and sequenced in a multimodal way (Kress and Selander, 2012). The use of multimodal pedagogies offers innovative possibilities for teachers to validate students’ literacies, experiences, and cultures, to support English language learning in the classroom (Kendrick et al., 2010). It is suggested for future studies to take into account the comparisons between the rural English teachers and urban English teachers from the perspective of multimodal discourse analysis, which can propose more specific suggestions on the improvements of the English teacher’s professional development in Chinese rural areas.

As for pedagogic implication, EFL teachers in Chinese rural areas need to be aware that multimodal competence plays a very important role in facilitating English classroom teaching in rural areas, which at some extent, can compensate the teachers’ English competence. Multimodal approach can also help students learn to exploit semiotic modes beyond verbal language (e.g., visual, gestural, spatial) to both understand and produce texts in the target language more effectively (O’Halloran, 2004). Therefore, this study suggests that when the teachers are accepted the professional development, teachers’ multimodal pedagogical awareness and multimodal competence need to be emphasized, which seems to be more important for English teachers in Chinese rural areas.

## Conclusion

This study has explored the multimodal analysis of English teaching delivered by four Chinese rural primary school English teachers. The findings reveal that English teachers in rural areas had a multimodal experience that happens through orchestration of spoken language and an array of other communicative modes, such as gesture, gaze, and facial expression, and AR is a useful method to improve teachers’ professional development. Through the observation and annotation of the four teachers’ English teaching videos before AR, five dimensions of their English teaching illustrate the features of rural primary school English teachers, all these can show those teachers need to improve their English teaching competencies. After Action Research which focused on pointing out their English teaching problems, as well as their English knowledge and usage, five dimensions of their English teaching changed in some way. Especially 2 months after AR, Teacher A designed and organized all the teaching content by herself, AR contributes greatly to reducing phonetic errors and grammatical errors; however, insignificant differences in three dimensions: gestures, intimate distance and facial expressions, which belonged to their teaching style. This justified that even after AR, as for those rural school English teachers, knowledge is easier to be corrected than their teaching style. More professional development needs to be done to improve their English teaching.

## Data availability statement

The raw data supporting the conclusions of this article will be made available by the authors, without undue reservation.

## Ethics statement

The studies involving human participants were reviewed and approved by Nantong University. The patients/participants provided their written informed consent to participate in this study.

## Author contributions

HZ and CH developed the study concept and contributed to the design. HZ, HM, and LW implemented the experiment and collected and analyzed the data. HZ, CH, and LW wrote and revised the manuscript. All authors contributed to the article and approved the submitted version.

## Funding

This work was supported by the National Social Science Foundation of China (19AYY021).

## Conflict of interest

The authors declare that the research was conducted in the absence of any commercial or financial relationships that could be construed as a potential conflict of interest.

## Publisher’s note

All claims expressed in this article are solely those of the authors and do not necessarily represent those of their affiliated organizations, or those of the publisher, the editors and the reviewers. Any product that may be evaluated in this article, or claim that may be made by its manufacturer, is not guaranteed or endorsed by the publisher.

## References

[ref1] Altrichter B. H. Al P. (1990). Teachers investigate their work: An introduction to the methods of action research. London and New York: Routledge.

[ref2] Barthes R. (1994). The rhetoric of the image in image-music-text. London: Fontana.

[ref3] Borich. (2002). Evaluation models: a question of purpose not terminology. Educ. Eval. Policy Anal. 5, 61–63. doi: 10.3102/01623737005001061

[ref4] Bowe J. Gore J. (2017). Reassembling teacher professional development: the case for quality teaching rounds. Teachers Teaching 23, 1–15. doi: 10.1080/13540602.2016.1206522

[ref5] Burns A. (1999). Collaborative action research for English language teachers. New York: Cambridge University Press.

[ref6] Cohen L. Manion L. Morrison K. (2000). Research methods in education. London: Routledge Falmer.

[ref7] Cope B. Kalantzis M. (2009). Multiliteracies: new literacies, new learning. Pedagogies: Intern. J. 4, 164–195. doi: 10.1080/15544800903076044

[ref8] Corder S. P. (1974). “Error analysis,” in Techniques in applied linguistics. eds. Allen J. P. B. Pit Corder S. (London: Oxford University Press).

[ref9] Crookes G. Chandler P. M. (2001). Introducing action research into the education of post-secondary foreign language teachers. Foreign Lang. Ann. 34, 131–140. doi: 10.1111/j.1944-9720.2001.tb02818.x

[ref10] Dai H. Legge J. (2016). The book of rites (in Chinese). Zhengzhou: Zhongzhou Classics Press.

[ref11] Davis B. Sumara D. Luce-Kapler R. (2000). Engaging minds: Changing teaching in complex times. London: Routledge.

[ref12] Dulfer N. Kriewaldt J. McKernan A. (2021). Using collaborative action research to enhance differentiated instruction. Int. J. Incl. Educ. 25, 1–15. doi: 10.1080/13603116.2021.1992678

[ref13] Gu Y. G. (2007). On multimedia learning and multimodal learning (in Chinese). Computer-assisted Foreign Lang. Educ. 4, 3–12. Available at: http://www.wydhjx.cbpt.cnki.net/WKG/WebPublication/paperDigest.aspx?paperID=6953fbcf-233e-427b-abd9-547e654f10dd

[ref14] Guo E. P. Gu C. M. Bao J. Y. (2002). Practice reports of “Sino-foreign teacher’s cooperation in their English teaching,” Programme (in Chinese). Foreign Lang. World 3, 47–52. Available at: https://kns.cnki.net/kcms/detail/detail.aspx?dbcode=CJFD&dbname=CJFD2002&filename=WYJY200203008&uniplatform=NZKPT&v=4oRKoEd98ZAF_xXKHYkDKQVDojU2E3IJRgMGEG6NU9SYx9FJ-O6ywGSZbwHip2_B

[ref15] Hu J. W. (2017). The application of multimodal teaching approach to English vocabulary teaching at rural junior middle school (in Chinese). Yili: Yili Normal University.

[ref16] Huang W. Ling J. (2016). Focus and value update: on the change of classroom management (in Chinese). Res. Educ. Develop. 36, 56–61. doi: 10.14121/j.cnki.1008-3855.2016.z2.011

[ref17] Jewitt C. (2006). Technology, literacy and learning: A multimodal approach. London and New York: Routledge.

[ref18] Kember D. (2000). Action learning and action research. London: Routledge.

[ref19] Kemmis S. McTaggart R. Nixon R. (1982). The action research planner. Victoria: Deakin.

[ref001] Kendrick M. Jones S. Mutonyi H. Norton B. (2010). Using drawing, photography and drama to enhance English language learning in Uganda. In Authenticity in the Language Classroom and Beyond: Children and Adolescent Learners. eds. Dantas-Whitney M. Rilling S. (Alexandria, VA: TESOL), 181–197.

[ref20] Kress G. R. (2003). Literacy in the new media age. London and New York: Routledge.

[ref21] Kress G. (2010). Multimodality: A social semiotic approach to contemporary communication. London: Routledge Falmer.

[ref002] Kress G. Jewitt C. Bourne J. Franks A. Hardcastle J. Jones K.. (2005). Urban English Classrooms. London: Routledge Falmer.

[ref003] Kress G. Selander S. (2012). Multimodal design, learning and cultures of recognition. Internet and Higher Education, 15, 265–268. doi: 10.1016/J.IHEDUC.2011.12.003

[ref22] Kress G van Leeuwen T. (1996). Reading images: The grammar of visual design. New York: Routledge.

[ref004] Lim F. V. (2021). Designing Learning with Embodied Teaching: Perspectives from Multimodality. London: Routledge.

[ref23] Long T. Zhao G. Yang X. Zhao R. Chen Q. (2021). Bridging the belief-action gap in a teachers’ professional learning community on teaching of thinking (in Chinese). Prof. Dev. Educ. 47, 729–744. doi: 10.1080/19415257.2019.1647872

[ref24] McNiff J. (1988). Action research: Principles and practice. London: Mcmillan Education.

[ref25] Ministry of Education (2022). National English Curriculum for compulsory education (in Chinese). Beijing: Beijing Normal University Press.

[ref26] Norris S. (2004). Analyzing multimodal interaction: A method of logical framework. London: Routledge.

[ref27] Norris S. (2020). Multimodal theory and methodology: For the analysis of (inter)action and identity. London: Routledge.

[ref28] Nunan D. (1990). Research methods in language learning. Cambridge: Cambridge University Press.

[ref29] O’Halloran K. L. (2004). Multimodal discourse analysis: Systemic-functional perspectives. Continuum. London: Routledge.

[ref30] OECD (1999). Education at a glance 1998: OECD indicators. Paris: OECD Publishing.

[ref31] OECD (2009). Creating effective teaching and learning environments: First results from TALIS. Paris: OECD Publishing.

[ref005] Peng J. (2019). The roles of multimodal pedagogic effects and classroom environment in willingness to communicate in English. System 82, 161–173. doi: 10.1016/j.system.2019.04.006

[ref32] Poortman C. Brown C. Schildkamp K. (2022). Professional learning networks: a conceptual model and research opportunities. Educ. Res. 64, 95–112. doi: 10.1080/00131881.2021.1985398

[ref33] Qiao Y. (2018). Observers’ behavior deviation on in-class observation and its correction (in Chinese). Educ. Res. 39, 104–108. Available at: https://kns.cnki.net/kcms/detail/detail.aspx?dbcode=CJFD&dbname=CJFDLAST2018&filename=JYYJ201810015&uniplatform=NZKPT&v=MIQ_TU5-kv2BbhTpysePEZiHc5dA9UIliUlzVOcMCAuPRLqFN_uSxgojCwT4aDMk

[ref34] Qin Y. Wang P. (2021). How EFL teachers engage students: a multimodal analysis of pedagogic discourse during classroom lead-ins. Front. Psychol. 12:793495. doi: 10.3389/fpsyg.2021.793495, PMID: 35002888 PMC8733723

[ref35] Romney. (1997). Collaborative learning in a translation course. Can. Mod. Lang. Rev. 54, 48–67. doi: 10.3138/CMLR.54.1.48

[ref36] Royce T. Terry D. (2015). Intersemiotic complementarity in legal cartoons: an ideational multimodal analysis. Int. J. Semiot. Law 28, 719–744. doi: 10.1007/S11196-015-9421-1

[ref37] Salman Z. M. Hazem A. H. Kamil D. F. Kanaan M. H. (2022). Teaching grammar to Iraqi EFL students of Al-Hamdaniya university during COVID-19 pandemic: problems and solutions. World J. English Lang. 12, 298–305. doi: 10.5430/wjel.v12n5p298

[ref38] Sun Y. Shi W. (2022). On the role of teacher–student rapport and teacher support as predictors of Chinese EFL students’ affective learning. Front. Psychol. 13:e856430. doi: 10.3389/fpsyg.2022.856430, PMID: 35360642 PMC8960135

[ref39] van Dijk W. Huizink A. C. Müller J. Uvnäs-Moberg K. Ekström-Bergström A. Handlin L. (2020). The effect of mechanical massage and mental training on heart rate variability and cortisol in swedish employees-a randomized explorative pilot study. Front. Public Health 8:e82. doi: 10.3389/fpubh.2020.00082, PMID: 32266197 PMC7098265

[ref40] Van Leeuwen T. (1999). Speech, music, sound. London: MacMillan.

[ref41] Wallace M. J. (1998). Action research for language teachers. Cambridge: Cambridge University Press.

[ref42] Wang L. Z. (2020). Application of multimodal teaching modes to English vocabulary teaching in rural junior middle schools (in Chinese). Lanzhou: Northwest Normal University.

[ref43] Wang Y. (2021). To be expressive or not: the role of teachers’ emotions in students’ learning. Front. Psychol. 12:e737310. doi: 10.3389/fpsyg.2021.737310, PMID: 35111095 PMC8802995

[ref44] Wang Y. Y. Wang Y. (2013). A study on the action research of college English listening curriculum reform from the perspective of circulation theory (in Chinese). Foreign Lang. Learning Theory and Practice 1, 49–54. Available at: https://kns.cnki.net/kcms/detail/detail.aspx?dbcode=CJFD&dbname=CJFD2013&filename=GWJX201301009&uniplatform=NZKPT&v=U-QXiB49h0Mb7Ud_CTgodPGn2ts8bjJeFy1SlkSuLJNjmf2IsPARKSIyrIbMS4Af

[ref45] Xia Y. Q. (2020). A study on the application of multimodality theory to English vocabulary teaching in rural junior high school (in Chinese). Nanjing: Nanjing Normal University.

[ref46] Yang H. (2019). An empirical study on the application of multimodal theory to English vocabulary teaching in rural primary schools (in Chinese). Xi’an: Xi’an International Studies University.

[ref47] Yang H. Li L. (2017). An action research on integration of intercultural competence with college English teaching (in Chinese). Foreign Lang. Their Teaching 2, 9–17. doi: 10.13458/j.cnki.flatt.004340

[ref48] Zhang D. (2009). On a synthetic theoretical framework for multimodal discourse analysis (in Chinese). Foreign Lang. China 1, 24–30. doi: 10.13564/j.cnki.issn.1672-9382.2009.01.004

[ref49] Zhang D. Wang Z. (2016). Theoretical framework for multimodal interaction analysis (in Chinese). Foreign Lang. China 13, 54–61. doi: 10.13564/j.cnki.issn.1672-9382.2016.02.008

[ref006] Zheng M. Chen F. (2000). The application of action research in reading instruction (in Chinese). Foreign Language Teaching and Research 32, 431–436. Available at: http://www.fltr.ac.cn/WKG/WebPublication/paperDigest.aspx?paperID=c5d66f2b-8cc1-4c8b-a77c-5f4d2e89b3e7

[ref50] Zhu D. Li P. (2015). Outline the effectiveness of classroom instruction (in Chinese). Educ. Res. 36, 90–97. Available at: https://kns.cnki.net/kcms/detail/detail.aspx?dbcode=CJFD&dbname=CJFDLAST2015&filename=JYYJ201510012&uniplatform=NZKPT&v=M9yMvAuC4wFAH9WdzzTvl3j5LRPnoWYGODTAjorEisBp8cvGWBMUeKJogdQ4_8N0

